# Trinor-cycloartane Glycosides from the Rhizomes of *Cimicifuga foetida*

**DOI:** 10.3390/molecules14041578

**Published:** 2009-04-17

**Authors:** Lu Lu, Jianchao Chen, Yin Nian, Yun Sun, Minghua Qiu

**Affiliations:** 1 State Key Laboratory of Phytochemistry and Plant Resources in West China, Kunming Institute of Botany, the Chinese Academy of Sciences, Kunming, 650204, P. R. China; 2 Graduate School of Chinese Academy of Sciences, Beijing 100039, P. R. China

**Keywords:** *Cimicifuga foetida*, Triterpenoids, Trinor-cycloartane, Glycosides

## Abstract

Three new trinor-cycloartane glycosides, 15α-hydroxy-16-dehydroxy-16(24)-en-foetidinol-3-*O*-*β*-d-xylopyranoside (**1**), 28-hydroxy-foetidinol-3-*O*-*β*-d-xylopyranoside (**2**) and foetidinol-3-*O*-*β*-d-xylopyranosyl-(1”→3’)-*β*-d-xylopyranoside (**3**) together with the known compound foetidinol-3-*O*-*β*-d-xylopyranoside (**4**) were isolated from the *n*-BuOH fraction of the roots of *Cimicifuga foetida*. Their structures were elucidated on the basis of spectroscopic and chemical reaction data.

## 1. Introduction

The Traditional Chinese Medicine Rhizoma Cimicifugea is used to treat toothache, mouth ulcers, sore throats and to help erupt measles [1]. The Chinese Pharmacopoeia describes three *Cimicifuga* species (*C. foetida*, *C. dahurica* and *C. heracleifolia*) as the crude drug source [1], among which *C. foetida* is found widely spread throughout northwest Yunnan Province [2].

**Figure 1:**
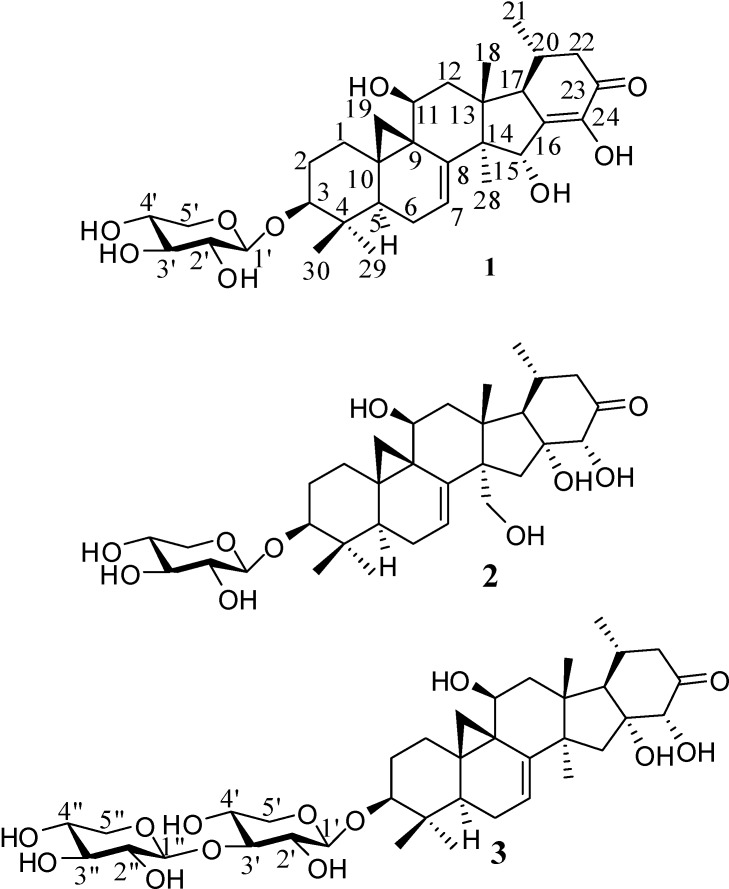
Structures of compounds **1**, **2** and **3**.

Ever since three trinor-cycloartanes were found in *C. foetida* by Asian research groups between 1994-96 [3,4,5,6,7], no additional compounds with such structure have been reported, as far as we know. While we were carrying out a phytochemical investigation on antitumor triterpenes of *Cimiciguga* species in Yunnan Province [8,9,10,11], three new trinor-cycloartane glycosides **1**-**3** were isolated from the roots of *C. foetida* (Figure 1).

## 2. Results and Discussion

**Figure 2:**
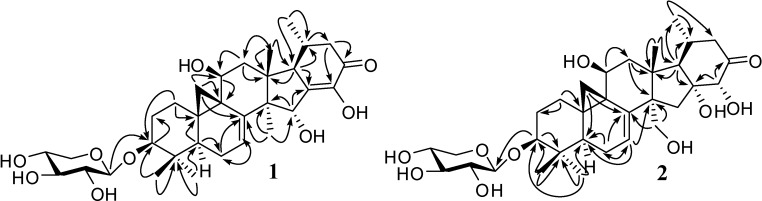
HMBC correlations of compounds **1** and **2**.

Compound **1**: colorless needles, [α${]}_{\text{D}}^{18}$-25.6° (c=0.14, MeOH). The [M-1]^-^ peak in the negative HRFABMS indicated a molecular formula of C_32_H_46_O_9_ (573.3014; calcd. for 573.3063). The IR spectrum displayed the presence of hydroxyl groups and a carbonyl group (ν_max_ 3426 br and 1671 cm^-1^). The UV data [(MeOH) λ_max_(logε) nm: 282(3.4), 321(3.4)] suggested the presence of an α,β-unsaturated ketone group. The ^13^C-NMR (Table 1) and DEPT spectra of **1** displayed 32 signals, of which 27 were attributed to a trinor-triterpene skeleton and five to a *β*-d-xylopyranose moiety [δ_C_ 67.2 t (C-5’), 71.3 d (C-4’), 75.6 d (C-2’), 78.7 d (C-3’), 107.6 d (C-1’)]. The trinor-triterpene structure included characteristic signals of five methyls [δ_C_ 14.6 (C-30), 19.4 (C-21), 20.3 (C-28), 20.5 (C-18), 26.0 (C-29)], three oxygenated methines [δ_C_ 63.5 (C-11), 76.5 (C-15), 88.5 (C-3)], an isolated olefinic bond [δ_C_ 115.3 (C-7), 147.4 (C-8)] and an enone system [δ_C_ 141.1 (C-16), 146.9 (C-24), 195.8 (C-23)]. The ^1^H-NMR spectrum of **1** (Table 1) showed five methyl signals at δ_H_ 0.86 (d, 6.43 Hz, 3H-21), 1.12 (s, 3H-18), 1.14 (s, 3H-30), 1.40 (s, 3H-29) and 1.47 (s, 3H-28), cyclopropane methylene protons at δ_H_ 1.01 (d, 3.70 Hz, H-19) and 1.97 (d, 3.79 Hz, H-19), three oxygenated methine protons at δ_H_ 3.59 (dd, 12.11, 3.65 Hz, H-3), δ_H_ 4.63 (dd, 9.35, 2.95 Hz, H-11), δ_H_ 5.36 (d, 2.90 Hz, H-15) and an olefinic hydrogen at δ_H_ 6.17 (dd, 7.80, 1.44 Hz, H-7). The chemical shifts assignable to rings A, B and C were similar to those of foetidinol-3-*O*-*β*-d-xyloside [3], except that an olefinic bond between C-16 and C-24 in **1** formed an enone group with the carbonyl group at C-23. The assignments were confirmed by the HMBC correlations of H-15 with C-14, C-16, C-24 and C-28, of H-22 with C-17, C-20, C-21, C-23 and C-24, and of H-17 with C-13, C-16, C-18 and C-20 (Figure 2). An α-orientation for OH-15 was deduced from the ROESY correlations of H-15 with 3H-18. Therefore, the structure was established as 15α-hydroxy-16-dehydroxy-16(24)-en-foetidinol-3-*O*-*β*-d-xylopyranoside.

Compound **2**: white powder, [α${]}_{\text{D}}^{19}$-62.0° (c=0.12, MeOH). The IR spectrum indicated the presence of hydroxyl groups, a carbonyl group and an olefinic bond (ν_max_ 3460 br, 1724 and 1636 cm^-1^). The [M-1]^-^ peak in the negative HRFABMS inferred a molecular formula of C_32_H_48_O_10_ (591.3131; calcd. for 591.3169). It has the same molecular formula as 15α-hydroxy-foetidinol-3-*O*-*β*-d-xyloside [3]. Comparison of its ^13^C-NMR data (Table 1) with those of 15α-hydroxy-foetidinol-3-*O*-*β*-d-xyloside, a methyl in the known compound was hydroxylated into a methylene at δ_C_ 67.4 in **2**, which was located at C-28 by the HMBC associations of H-28 with C-8, C-12 and C-14, H-12 with C-13, C-14, C-17 and C-28. In addition, the other four methyls had no possibility to be hydroxylated due to 3H-21 and 3H-18 showed HMBC correlations with C-17 while 3H-29 and 3H-30 correlated with C-3, C-4 and C-5. Thus, the structure of **2** was elucidated as 28-hydroxy-foetidinol-3-*O*-*β*-d- xylopyranoside.

**Figure 3:**
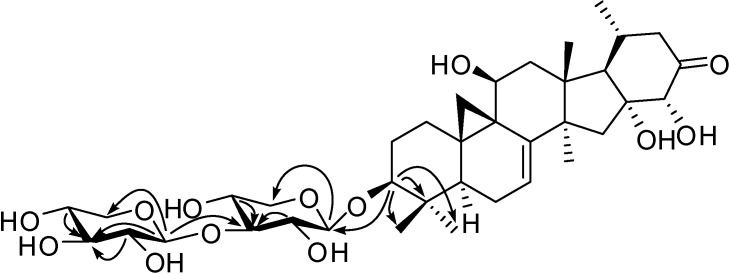
HMBC correlations of the disaccharide in compound **3**.

Compound **3**: white powder, [α${]}_{\text{D}}^{19}$-46.2° (c=0.12, C_5_H_5_N). The IR spectrum indicated the presence of hydroxyl groups, a carbonyl group and an olefinic bond (ν_max_ 3410 br, 1724 and 1633 cm^-1^). The [M+Cl]^-^ peak in the negative HRESIMS corresponds to a molecular formula of C_37_H_56_O_13_ (743.3393; calcd. for 743.3409). The NMR data (Table 1) of the aglycon moiety were in good agreement with those of foetidinol aglycone [3], however, the ten additional resonances in the ^13^C-NMR spectrum at δ_C_ 66.6, 67.5, 69.4, 71.0, 74.5, 75.4, 78.3, 87.4, 106.3 and 107.2 and the signals of two anomeric protons at δ_H_ 4.82 (d, 7.49 Hz, H-1’) and 5.27 (d, 7.66 Hz, H-1”) in ^1^H-NMR spectrum indicated the existence of two pentoses in **3**. The ^13^C-NMR data of the pentoses revealed the presence of xylose, and in the ROESY spectrum, H-1’ showed associations with H-3’ and H-5’ (δ_H_ 3.70 m) while H-1” showed associations with H-3” and H-5”. This was confirmed by acid hydrolysis and TLC comparison with an authentic sample. A linkage of the diglycoside of xylosyl-(1”→3’)-xylosyl to C-3 of the aglycone was proved by the HMBC correlations of H-3 with C-1’, C-4, C-29 and C-30; of H-1’ with C-3 and C-5’, of H-1” with C-3’, C-3” and C-5”; of H-2’, H-4’, 2H-5’ and H-1” with C-3’ and of H-2”, H-4” and 2H-5” with C-3” (Figure 3). In sum, compound **3** was established as foetidinol-3-*O*-*β*-d-xylopyranosyl-(1”→3’)-*β*-d-xylopyranoside.

**Table 1:** ^1^H- and ^13^C-NMR Data for Compounds **1, 2** and **3**.

	1				2				3			
No.	δ_C_		δ_H_, mult. (J in Hz)		δ_C_, mult.		δ_H_, mult. (J in Hz)		δ_C_, mult.		δ_H_, mult. (J in Hz)	
	C_5_D_5_N											
1	27.6 t		1.72m		27.5 t		1.61 t (12.2)		27.5 t		1.67 m	
			2.81 m				2.62 d (12.9)				2.72 dt (13.7, 3.6)	
2	30.0 t		2.10 m		29.9 t		2.03 m		29.9 t		2.05 m	
			2.41 m				2.35 m				2.33 m	
3	88.5 d		3.59 dd (12.1, 3.7)		88.4 d		3.47 dd (11.1, 3.0)		88.8 d		3.55 dd (11.9, 4.1)	
4	40.8 s				40.7 s				40.8 s			
5	44.0 d		1.43 m		43.8 d		1.40 m		44.1 d		1.35 dd (12.6, 4.9)	
6	22.1 t		1.76 m		22.2 t		1.76 dd (27.0, 13.5)		22.1 t		1.76 m	
			2.03 m				1.92 dd (16.4, 6.1)				1.94 m	
7	115.3 d		6.17 dd (7.8, 1.4)		117.5 d		5.26 d (7.0)		113.8 d		5.20 dd (7.8, 1.6)	
8	147.4 s				144.4 s				149.4 s			
9	27.8 s				27.6 s				27.5 s			
10	29.1 s				29.3 s				29.2 s			
11	63.5 d		4.63 dd (9.4, 3.0)		63.5 d		4.57 m		63.6 d		4.59 m	
12	48.1 t		2.02 m		44.0 t		2.46 m		49.0 t		2.06 m	
			2.84 m				2.80 d (13.8)				2.84 dd (14.0, 9.6)	
13	43.6 s				46.5 s				46.4 s			
14	53.2 s				56.8 s				50.9 s			
15	76.5 d		5.36 d (2.9)		47.9 t		2.17 m		48.6 t		2.25 m	
							3.00 dd (13.3, 9.4)				2.53 m	
16	141.1 s				82.4 s				82.1 s			
17	54.9 d		2.55 dd (10.1, 2.9)		64.1 d		2.40 m		63.7 d		2.21 m	
18	20.5 q		1.12 s		21.7 q		1.34 s		21.3 q		1.26 s	
19	18.6 t		1.01 d (3.7)		19.2 t		1.02 d (2.8)		18.8 t		1.01 d (3.5)	
			1.97 d (3.8)				2.02 d (3.6)				1.98 d (3.8)	
20	34.2 d		1.90 ddt (9.7, 6.6, 3.3)		26.0 d		2.20 m		25.9 d		2.17 m	
21	19.4 q		0.86 d (6.4)		20.7 q		0.88 d (5.9)		20.8 q		0.91 d (6.1)	
22	47.1 t		2.20 dd (15.8, 2.2)		45.0 t		2.46 m		45.0 t		2.41 dd (18.8, 3.2)	
			2.51 dd (16.4, 3.6)				2.46 m				2.48 d (12.3)	
23	195.8 s				211.3 s				211.5 s			
24	146.9 s				82.3 d		4.58 s		82.5 d		4.49 s	
28	20.3 q		1.47 s		67.4 t		3.77 d (6.7)		28.2 q		1.59 s	
							4.47 d (10.8)					
29	26.0 q		1.40 s		25.9 q		1.33 s		25.9 q		1.39 s	
30	14.6 q		1.14 s		14.6 q		1.12 s		14.7 q		1.14 s	
1’	107.6 *d*		4.88 d (7.5)		107.6 *d*		4.85 d (7.4)		107.2 d		4.82 d (7.5)	
2’	75.6 *d*		4.03 t (8.1)		75.6 *d*		4.02 t (7.8)		74.5 d		4.03 m	
3’	78.7 *d*		4.16 t (8.7)		78.7 *d*		4.17 dd (8.2, 16.8)		87.4 d		4.11 m	
4’	71.3 *d*		4.20		71.3 *d*		4.22 dd (11.7, 6.7)		69.4 d		4.08 m	
5’	67.2 *t*		3.73 dd (10.9,10.1)		67.2 *t*		3.73 dd (10.2, 6.2)		66.6 t		3.70 m		
			4.34 dd (11.3, 5.0)				4.35 dd (11.2, 4.7)				4.29 m	
1”									106.3 d		5.27 d (7.7)		
2”									75.4 d		4.01 m		
3”									78.3 d		4.14 m		
4”									71.0 d		4.15 m		
5”									67.5 t		3.66 m		
											4.29 m		

## 3. Experimental

### 3.1. General

Optical rotations: Horiba SEPA-300 polarimeter. UV spectra: Shimadzu UV-2401A spectrophotometer. IR spectra: Bio-Rad FTS-135 infrared spectrophotometer. ^1^H-, ^13^C-NMR and 2D-NMR spectra: Bruker AM-400 MHz or DRX-500 spectrometers with TMS as internal standard. MS: VG Autospec-300, Finnigan MAT-90 and API Qstar-Plusar-1 spectrometers. The pentose authentic samples were purchased from Acros Organics.

### 3.2. Plant material

The roots of *C. foetida* were collected in Daju Village of Lijiang County, Yunnan Province in July 2004 and identified by Prof. Pei Shengji (Kunming Institute of Botany, the Chinese Academy of Sciences). The voucher specimen (KIB 04072601) has been deposited at the State Key Laboratory of Phytochemistry and Plant Resources in West China, Kunming Institute of Botany, Chinese Academy of Sciences.

### 3.3. Extraction and Isolation

Air-dried and powdered roots of *C. foetida* (10 kg) were extracted three times with MeOH (25 L) under reflux. After removal of the solvent by evaporation, the residue (950 g) was suspended in H_2_O and partitioned sequentially with CHCl_3_ and *n*-BuOH. The *n*-BuOH fraction (250 g) was subjected to silica gel chromatography and eluted with CHCl_3_-MeOH (20:1, 10:1, 8:1, 5:1) to give four fractions (Fr. A - D). Fr. B (20 g) was chromatographed repeatedly over RP-18 (45-70% MeOH-H_2_O) to successively yield compounds **2** (8 mg), **4** (200 mg), **1** (20 mg) and **3** (25 mg).

*15 α-Hydroxy-16-dehydroxy-16(24)-en-foetidinol-3-O-β-d-xylopyranoside* (**1**): colorless needles; [α${]}_{\text{D}}^{18}$ - 25.6° (c=0.14, MeOH); HRFABMS (573.3014; calcd for C_32_H_45_O_9_, 573.3063). IR (KBr) cm^-1^: 3426 (br, OH), 1671 (C=O); UV (MeOH) λ_max_ (logε) nm: 282 (3.4), 321 (3.4); ^1^H- and ^13^C-NMR data, see Table 1.

*28-Hydroxyfoetidinol-3-O-β-d-xylopyranoside* (**2**): white powder, [α${]}_{\text{D}}^{19}$ - 62.0° (c=0.12, MeOH); HRFABMS (573.3131; calcd for C_32_H_48_O_10_, 573.3169). IR (KBr) cm^-1^: 3460 (br, OH), 1724 (C=O), 1636 (C=C); ^1^H- and ^13^C-NMR data, see Table 1.

*Foetidinol-3-O-β-d-xylopyranosyl-(1”→3’)-β-d-xylopyranoside* (**3**): white powder, [α${]}_{\text{D}}^{19}$ - 46.2° (c=0.12, C_5_H_5_N); HRESIMS (743.3393; calcd for C_37_H_56_O_13_Cl, 743.3409). IR (KBr) cm^-1^: 3410 (br, OH), 1724 (C=O), 1633 (C=C); ^1^H- and ^13^C-NMR data, see Table 1.

### 3.4. Acid hydrolysis of compounds 1-3

Compounds **1**-**3** (2 mg of each) were refluxed with 6% HCl-MeOH-*n*-BuOH (20 mL, 2:1:1 *v*/*v/v*) for 1 h at 90 °C, then neutralized with 12 M NaOH. The concentrated methanol soluble part showed a TLC spot (*n*-BuOH-acetone-H_2_O, 4:3:1, *Rf* = 0.7) matching that of an authentic sample of d-xylose.

## 4. Conclusions

Since three trinor-cycloartanes were found for the first time in *C. foetida* between 1994 and 1996, no such type of structure have been reported in past ten years. The new compounds found were trace constituents compared with foetidinol-3-*O*-*β*-d-xylopyranoside, and presumably are formed as side products or intermediates due to elimination, oxygenation or glycosylation reactions during the biosynthesis of this compound [12]. They suggest a diversity of trinor-cycloartane structures and the high-content of foetidinol-related structures found in Daju village of Lijiang County made the *C. foetida* growing in this area a new resource of novel trinor-cycloartane structures.
